# The physicochemical properties and antioxidative potential of raw thigh meat from broilers fed a dietary medicinal herb extract mixture

**Published:** 2014-07-11

**Authors:** K. Shirzadegan, P. Falahpour

**Affiliations:** 1 *Department of Animal Science, Faculty of Agriculture, University of Zanjan, Zanjan, Iran*; 2 *Department of Animal Science, Faculty of Agriculture and Natural Resources, Khorasgan Branch, Islamic Azad University, Khorasgan, Iran*

**Keywords:** Antioxidative component, Broilers, Extract mixture, Meat

## Abstract

A 6-wk feeding study was conducted to evaluate the antioxidative potential, indices such as quality of the thigh meat and liver of broiler chickens fed with a dietary medicinal herb extract mixture (HEM, consisting: Iranian green tea, cinnamon, garlic and chicory at a ratio of 25:15:45:15). A total of 320, one-d-old Ross (male) broiler chickens were used to investigate the effects of 0.0, 2.5, 5.0 and 7.5 g/kg HEM in the diet, on aforementioned factors. The HEM supplementation did not influence the composition of raw thigh meat except for the total phenols and crude ash (*P*<0.05). Furthermore, pH, water-holding capacity (WHC) and acceptability of thigh meat were affecting by administration of HEM in diets (*P*<0.05). Meat flavor increased in the supplemented groups (*P*<0.05). According to our data, HEM supplementation decreased the amount of thiobarbituric acid reactive substance (TBARS) in various times of storage and improved the liver lipid peroxides and superoxide dismutase (SOD) activities at week 6 (*P*<0.05), but did not influence the catalase activity. Our results reveal that the addition of 7.5 g/kg or higher HEM in diet could be sufficient to increase the antioxidative activity and 2.5 g/kg for meat taste of broilers in maximum levels.

## Introduction

With the development and wide use of synthetic and semi-synthetic antibiotics during last 50 years, researches look back to natural products as indispensable resources (Ferrini *et al.*, 2008). Extracted oils from plants have been previously used to test the effect on broilers growth performance (Antar *et al.*, 2004). One of these herbs is chicory (*Cicorium intybus L*.), a perennial herb used as a palatable forage crop for sheep, deer, and cattle (Li and kemp, 2005).

The chicory forage has a high content of uronic acids, which in dicotyledonous plants are derived from galactosyl uronic acid. Uronic acid is the building block in pectin (Voragen *et al.*, 2001). Chicory root has a high content of fructo oligosaccharides and inulin, which can be used to manipulate the composition of microflora in the gut and enhance its integrity (Flickinger *et al.*, 2003). Inulin is one of the best-studied prebiotic sources in domestic animal applications (Castellini *et al.*, 2007; Attia *et al.*, 2014). Thus, both the forage and the root of chicory are of interest as fiber sources in poultry nutrition.

Green tea is a historically popular beverage among Asian population, and is produced from the leaves of the evergreen plant *Camellia sinensis*. Several studies have reported on green tea by-products that are used as potential sources of natural antioxidants or functional materials (Lee *et al.*, 2006). The major active ingredients in green tea are polyphenolic compounds known as catechins (Balentine *et al.*, 1997), which are reported to possess antiviral and antimicrobial activities (Yan *et al.*, 2004).

Studies show that oxidative damage occurs in animals due to an imbalance between the production of reactive oxygen or nitrogen species and the defense mechanism of the animal against oxidative stress (Morrissey *et al.*, 1998). Oxidation increases as a result of a high intake of oxidized lipids, oxidation of sensitive polyunsaturated fatty acids (PUFA) or prooxidants, or a low intake of nutrients involved in the antioxidant defense system (Morrissey *et al.*, 1998). An increasing number of studies have reported on the antioxidant properties of herb extracts and compounds in vitro or when added during food processing (Pellegrini *et al.*, 2003).

Cinnamon (*Cinnamomum verum*) is another herb commonly used as a spice in human food and has received considerable attention as an additive in poultry nutrition. It is reported that, cinnamon has an antioxidant activity and it also improved meat quality of broilers (Ciftci *et al.*, 2010).

Hernandez *et al*. (2004) showed that 200 ppm essential oil extract from oregano, cinnamon and pepper improved nutrient digestibility in broilers. Moreover, the antibacterial activity of *cinnamaldehyde* and the essential oil obtained from cinnamon leaves was demonstrated earlier (Chen *et al.*, 2008).

Garlic (*Allium sativum*) is also a common plant that grows on rural sites, in gardens, at the edges of forests and in wooded areas of rivers. In the past two decades, particular attention has been focused on the cholesterol-lowering activity of garlic. Bordia *et al*. (1975) reported that the essential oils of onion and garlic can prevent fat-induced hyperlipemia.

A marked reduction of serum cholesterol levels were observed in rats fed a diet supplemented with 20 or 30 g/kg garlic powder (Bordia *et al.*, 1975). Similar effects of garlic were found in rats fed diets containing either cholesterol or lard (Bordia *et al.*, 1975). Therefore, the aim of this study was to evaluate the antioxidative potential, quality of the thigh meat and liver indices of broiler chickens fed a dietary medicinal herb extract mixture (HEM).

## Materials and Methods

### Experimental design and management

All research protocols were reviewed and approved by the Animal Care Committee of Khorasgan University, Iran. In a completely randomized design with 3 treatments and a control group, 320 one-day-old male broiler chicks (Ross 308, mean weight ± 40g) were provided by a commercial local hatchery and housed in 16 pens (2 m^2^) of 20 birds each, weighed, wing banded, and assigned to pens in stainless steel chick batteries.

Chicks were maintained on a 24-h continuous light schedule and allowed ad libitum access to feed and water during the 42-d study. The temperature was 32°C during the first 2 d, and then reduced by 2°C weekly until 35 d, and then the temperature was kept constant at 22°C until the end of the rearing period.

The corn-soybean meal based basal diets (mash form) contained: crude protein 230.0 g/kg, ME 13.38 MJ/kg (starter); or crude protein 200.0 g/kg, ME 13.38 MJ/kg (grower). The experimental design allocated the groups as follows: 1. an unsupplemented diet (control); 2, 3 and 4: the diet supplemented with the medicinal herbal extract mixture (HEM) derived from, green tea, Cinnamon, garlic, and chicory at a ratio of (25:15:45:15) at a level of 2.5, 5.0 and 7.5 grams per kg of feed.

For the treatments, fresh herbs were harvested from a resource garden in Iran. The herbs were chopped and pulverized to pass 100 meshes (2.5 mm). An extract of the medicinal herb was prepared with 70% methanol. Then the dose titrations were achieved by addition of 0.0. 2.5, 5.0 and 7.5 g medicinal herb extract mix per kg diet at the expense of washed builder’s sand, till it provided four isocaloric and isonitrogenous diets (Table 1). The diets were formulated (mash form) to meet exactly the nutritional requirements of chicks from hatch to 6 weeks as recommended by the NRC (1994).

**Table 1:** Composition of broilers starter and grower diets.

	Diets^1^			
Ingredients (g/kg)	Control	2.5	5.0	7.5
Soybean meal-44	374.^2^ (332.2)^3^	273.4 (232.2)	272.2 (232.3)	271.6 (233.4)
Wheat, white W-	150 (150)	150 (150)	150 (150)	150 (150)
Corn, Grain	253.3(334.4)	253.3 (331.3)	253.5 (331.3)	253.6 (330.0)
Barley, Pacific	100 (100)	100 (100)	100 (100)	100 (100)
Bakery waste	100 (100)	100 (100)	100 (100)	100 (100)
Corn oil	70 (60.5)	70 (60.6)	70 (60.8)	70 (60.9)
Anchovy meal	20 (0)	28.4 (0)	29.2 (0)	29.6 (0)
Dical-phos	13.8 (12.1)	12.2 (9.31)	5.2 (3.64)	13.9 (0.8)
Oyster shells	11.7 (13.8)	11.0 (12.1)	13.2 (14.3)	11.7 (15.3)
Min+Vit^4^	5.0 (5.0)	5.0 (5.0)	5.0 (5.0)	5.0 (5.0)
Salt	3.0 (3.0)	3.0 (3.0)	3.0 (3.0)	3.0 (3.0)
HEM	0 (0)	2.5 (2.5)	5.0 (5.0)	7.5 (7.5)
sand	7.5 (7.5)	5.0 (5.0)	2.5 (2.5)	0 (0)
DL-Methionine	0.96 (0.86)	0.95 (0.86)	0.96 (0.86)	0.96 (0.86)
Total	1000	1000	1000	1000
Analyzed				
ME (kcal/kg)	3200 (3200)	3200 (3200)	3200 (3200)	3200 (3200)
CP (g/kg)	230.0 (200.0)	230.0 (200.0)	230.0 (200.0)	230.0 (200.0)
Ca (g/kg)	10.0 (9.0)	10.0 (9.0)	10.0 (9.0)	10.0 (9.0)
Available phosphorus (g/kg)	4.5 (3.5)	4.5 3.5 (3.5)	4.5(3.5)	4.5 (3.5)
Sodium (g/kg)	2.0 (1.5)	2.0 (1.5)	2.0 (1.5)	2.0 (1.5)

1 Control = 0.0 g/kg medicinal herb extract (HEM, consisting of Iranian green tea, cinnamon, garlic and chicory at a ratio of 25:15:45:15); T1 = 2.5 g/kg HEM; T2 = 5.0 g/kg HEM, and T3 = 7.5 g/kg HEM. The dose titrations were achieved by addition of HEM at the expense of washed builder’s sand.

2 Starter diet (0 to 21 days old).

3 Grower diet (22 to 42 days old).

4 The vitamin-mineral premix contained for starter and grower periods (per kg of diet): retinol, 10 000 IU; cholecaciferol, 1000 IU; menadione, 2.5 mg; cyanocobalamin, 21 mg; riboflavin, 4.8 mg; pantothenate, 42 mg; niacin, 22 mg; choline, 815 mg; biotin, 32 mg; thiamine, 4 mg; riboflavin, 6.6 mg; pyridoxine, 3 mg; folic acid, 1 mg; manganese (magnesium oxide), 60 mg; zinc (zinc sulphate), 60 mg; selenium (sodium selenite), 0.14; iron (ferrous sulphate), 20 mg; magnesium (magnesium oxide), 12 mg; iodine (calcium iodate), 0.80 mg; copper (cupric sulphate), 0.81 mg.

The contents of polyphenol, protein were determined by a spectrophotometer using the following methods: polyphenol by ferrum tartrate method, protein by Coomassie brilliant blue method, total sugar by anthrone reagent method (Zhong, 1989), crud fat by Soxhlet extractor. The moisture and total ash contents were determined by the methods described in AQS (2002a, b).

At the end of the experimental period (42 days), broilers were fasted for 6 h. 2 birds were randomly collected from each pen, and then were transferred to the slaughterhouse and treated with conventional procedures. Broilers were slaughtered by neck cut according to Islamic method.

After slaughter, thigh muscles were isolated from the carcasses. All skin along with the fat and visible connective tissues was removed from the thigh muscles before evaluation for different quality parameters. Also, a liver sample was collected of each bird. The samples (thighs and livers) were stored at −75°C for determination of thiobarbituric acid reactive substances (TBARS) and liver enzymes.

For measuring of thiobarbituric acid-reactive substances (over the 0, 5 and 10 days of storage); in first, two right thighs originating from each broiler were removed. Each meat sample (5 g) from various storage periods was homogenized in 15 mL of distilled water. Sample homogenate (5 mL) was transferred to a test tube and lipid oxidation was determined as the TBARS value by using the method described by (Ahn *et al.*, 1999).

Briefly, 50 μL of butylated hydroxyanisol (7.2%) and 5 mL of TBA-trichloroacetic acid (TCA) solution (20 mM TBA in 15% trichloroacetic acid) were added to the test tube. Tubes were heated in a boiling water bath for 18 min, cooled, and then centrifuged at 1000 × g for 15 min. Absorbance of the supernatant was measured at 540 nm with a spectrophotometer (UV 1600 PC, Heidari). The increase in absorbance at 540 nm was considered to calculate the TBARS values. Lipid oxidation was reported as mg of malondialdehyde per kg of thigh meat.

Liver tissue samples were also diluted with ice-cold phosphate buffered saline (pH=7.2) without heparin at a ratio of 1:9, homogenized in a homogenizer (Tekmar, SDT 1810, Cincinnati, OH), and centrifuged (10,000 × g, 4°C, 20 min). The clear supernatant was aspirated into vials and preserved in different aliquots at −70°C until antioxidant status was determined. The parameters measured included total lipid peroxide, superoxide dismutase (SOD), and catalase using the assay kits (Sigma Diagnostics, Sigma Chemical Co., H. Heidari). The muscle pH was measured using a digital pH meter (Model 520A, Orion, Beverly, MA).

For calculating the cooking loss and water-holding capacity (WHC), frozen thigh samples were thawed at 5°C overnight. The thawed thighs were weighed and baked in an oven (General Electric, Iran) to a final internal temperature of 77° C. Then the cooked thighs were cooled to an ambient temperature (20°C), patted dry with 1 paper towel (2-ply), and reweighed. Cooking loss was reported as a g/kg and calculated as: (initial weight – final weight).

For measuring WHC, 10 g of ground chicken meat was introduced to a centrifugal pipe and heated for 30 min at 70°C in a water bath. Then, it was cooled to room temperature and centrifuged at 5°C at low speed (1,000 for 10 min). Drip loss was indicated as g/kg of samples.

The sensory panels consisting of students, faculty, and staff of Khorasgan University were used to evaluate the sensory properties of the chicken thigh meat. Panelists (50% untrained and 50% trained, randomly chosen individuals) were selected based on their frequency of meat consumption and willingness to participate in the test.

Although untrained, most of the panelists were almost familiar with sensory testing from other studies. Cooked chicken thigh pieces from each treatment were placed in covered bowl containers and served warm (40°C) to each panelist one at a time. Panelists evaluated a total of 5 samples every week. The samples were transferred into bowl containers (Pyrex type) covered with polyethylene cap about 20 min before the sensory test started. The panelists (5 per repeat) evaluated the samples for hardness, juiciness, flavor and acceptability using a 5-point hedonic scale as described by (Carr *et al.*, 1999).

A score of 1 represented attributes most disliked and a score of 5 represented attributes most liked. All of the output results are reported by means.

### Statistical analysis

All data were analyzed by ANOVA using the GLM procedure of SAS (version 8.2, 2002). Duncan’s multiple range test (1955) was used to determine the statistical significance among the means at a 95% significance level. As the following model:

Yij= µ+Ti+ e_i j_

Where Yij was the amount of each observation, µ is the overall mean, Ti is the effect of the treatments and e_i j_ is the experimental error.

## Results

### Proximate composition

Table (2) shows the effect of the dietary supplementation with different levels of HEM on proximate composition of chicken thigh muscle. Moisture, crude protein and crude fat did not show significant differences among chicken samples (*P*>0.05). However, dietary supplementation with HEM resulted in significantly greater total phenols content linearly (y = 54.44 + 4.192 x; *P*<0.05).

**Table 2:** Proximate composition (g/kg) and total phenols content (μg/g) of raw thigh meat from chickens^1^ fed a dietary medicinal HEM for 42 d.

HEM^2^(g/kg)	Total phenol (ppm)	Crude protein (g/kg)	Crude fat (g/kg)	Moisture (g/kg)	Crude ash (g/kg)
0.0	51.292^d^	171.75	26.776	748.31	4.940^a^
2.5	68.371^c^	161.82	23.588	739.36	3.712^b^
5.0	77.956^b^	176.39	21.383	743.40	3.339^b^
7.5	83.030^a^	180.10	21.625	750.14	2.609^c^
Pooled SEM	0.73	5.5	0.8	2.2	0.06
Linear	0.03	0.49	0.36	0.52	0.11
Quadratic	0.02	0.15	0.24	0.08	0.06

^a-d^Means within a columns with different letters superscripts are significantly different (P<0.05).

1 All measurements were performed on 2 chickens per pen.

2 HEM = herbs extract mixture.

### Physicochemical and sensory analyses

The effect of the dietary supplementation of different levels of HEM on pH, cooking loss, WHC, juiciness, hardness, flavor and consumer acceptability in experimental chickens showed in (Table 3).

**Table 3:** Influence of dietary supplementation with HEM on pH, WHC, cooking loss, juiciness, hardness, flavor and acceptability of broiler chicken^1^ thigh muscle.

HEM^2^ g/kg	pH	WHC g/kg	Cooking loss g/kg	Juiciness	Hardness	Flavor	Acceptability
0.0	5.895^a^	629.0^a^	236.5	4.184	4.261^ab^	4.268^b^	4.107^c^
2.5	5.758^ab^	616.1^ab^	231.6	4.301	4.360^ab^	4.505^a^	4.462^a^
5.0	5.764^ab^	573.7^b^	227.7	4.270	4.257^b^	4.513^a^	4.376^ab^
7.5	5.661^b^	586.2^ab^	216.9	4.344	4.486^a^	4.409^ab^	4.225^bc^
Pooled SEM	0.02	6.6	4.0	0.04	0.03	0.03	0.02
Orthogonal contrast							
Control vs. others	0.009	0.037	0.089	0.17	0.052	0.024	0.009
2.5 × 5.0 × 7.5	0.48	0.77	0.31	0.06	0.44	0.13	0.024
Linear	0.31	0.09	0.02	0.45	0.66	0.54	0.56
Quadratic	0.25	0.28	0.56	0.33	0.21	0.09	0.07

^a,b^Means within columns with different superscripts are significantly different (P<0.05).

1 Means represent 2 chicks per pen.

2 HEM = herbs extract mixture.

In this study, broiler diet supplementation with 7.5 g/kg HEM resulted in a lower pH value in thigh muscle compared with control (*P*<0.05). There was an almost linear decrease in pH with increasing levels of dietary HEM, which was significantly lower in the 7.5 g/kg treatment. However, the muscle's pH value ranged from 5.66 (in supplemented sample) to 5.89 (in control sample), which all pH values were within the range expected for normal chicken.

A significant difference was found in the WHC of thigh meat in the end of the experimental period (Table 3).

The highest and the lowest levels were related to 0.0 and 5.0 g/kg HEM respectively (*P*<0.05). In the same line, once thigh meat was cooked, the taste intensity (flavor and acceptability) was significantly higher in 2.5 g/kg (*P*<0.05).

There were no significant differences, but supplemented diets showed higher values for juiciness (*P*>0.05), and differences were significant for hardness (*P*<0.05). Moreover, in this study, no significant differences were found in cooking loss among the chicken meat samples for diets with different levels of HEM.

Except for the significant (*P*<0.05) interaction among control vs. other treatments on cooking loss and juiciness, other interactions were not significant. Also, no significant interaction was observed between the 2.5, 5.0 and 7.5 g/kg of HEM, except to acceptability (*P*<0.05) (Table 3).

### Lipid oxidation (TBARS)

There was a significant difference in TBARS between broiler thigh meat due to diet at each storage time (0, 5 and 10 days). However, there was a trend for TBARS to have a greater increase over time for all treatments, but the broilers that achieved HEM (2.5 to 7.5 g/kg) had less TBARS as compared to control group. The equations in the 0 d of storage was (y = 0.60 – 0.027 x; *P*<0.05) as dietary HEM increased.

The interaction of control × other treatments and various levels of HEM, was significant on TBARS duration of storage times (Table 4).

**Table 4:** TBARS of raw thigh meat from chickens1 fed a dietary medicinal herb extract mix (HEM) for 42 d.

TBARS^2^ (mg)	Storage times		
	d- 0	d- 5	d- 10
0.0 HEM^3^	0.602^a^	0.780^a^	0.926^a^
2.5 HEM	0.566^b^	0.591^b^	0.631^eb^
5.0 HEM	0.453^c^	0.497^c^	0.609^b^
7.5 HEM	0.410^d^	0.485^c^	0.587^c^
Pooled SEM	0.005	0.003	0.003
Orthogonal contrast			
Control vs. others	0.0001	0.0001	0.0001
2.5 × 5.0 × 7.5	0.0001	0.0001	0.0002
Linear	0.02	0.10	0.06
Quadratic	0.07	0.08	0.27

^a,b^Means within a column with different letters are significantly different (*P*<0.05).

1 All measurements were performed on 2 chickens per pen.

2 TBARS values of the all treatments were significantly increased by storage, (mg of malondialdehyde per kg of meat).

3 HEM = herbs extract mixture (g/kg).

HEM supplementation linearly decreased the TBARS of thigh of broilers in week 1, whereas no significant linear reduction in data was observed after this point. Total TBARS (mean values of 0, 5 and 10 d) decreased quadratically (y = 0.75 – 0.08 x + 0.0059 x^2^; *P*<0.05; R^2^ = 0.98) as dietary HEM level increased.

### Liver antioxidant status

Dietary HEM supplementation had no effect on liver catalase activity at week 6 (Table 5), but liver SOD was increased linearly (y = 0.69 + 0.022 x; *P*<0.05) as dietary HEM increased. Furthermore, serum catalase activity decreased (*P*>0.05) quadratically at week 6 as dietary HEM level increased (y = 28.15 − 1.359 x + 0.143 x^2^; *P*<0.05; R^2^ = 0.99).

**Table 5:** Antioxidant status in liver of chicks^1^ fed diets containing of different levels HEM as natural antioxidants.

HEM^2^ g/kg	Lipid peroxides (µM/mL)	SOD^3^ (U/mg of protein)	Catalase^4^ (U/mg of protein)
0.0	0.322^a^	0.717^c^	28.120
2.5	0.280^b^	0.725^c^	25.753
5.0	0.303^ab^	0.809^b^	24.851
7.5	0.244^c^	0.876^a^	26.074
Pooled SEM	0.005	0.006	0.77
Orthogonal contrast			
Control vs. others	0.002	0.0001	0.44
2.5 × 5.0 × 7.5	0.07	0.0001	0.09
Linear	0.22	0.03	0.13
Quadratic	0.18	0.08	0.05

^a,b,c^Means with different superscripts in a column differ significantly (*P*<0.05).

1 Means represent 2 chicks per pen.

2 HEM = herbs extract mixture.

3 SOD = superoxide dismutase; one unit of activity is the amount of enzyme that inhibits the rate of reaction by 100 percent.

4 One unit of activity is the amount of enzyme that degrades 1 µM of hydrogen peroxide per min at 25°C.

Lipid peroxide level also increased in liver homogenate of chicks fed control and supplementation of the basal diet with HEM reduced (*P*<0.05) peroxide levels (Table 5). The lowest lipid peroxide was observed in 7.5 g/kg group. The interaction of control × other treatments and various levels of HEM (except to lipid peroxides), was significant for only SOD, but no significant difference was observed for catalase (mmol/L).

## Discussion

This experiment showed that, supplementation with HEM did affect the proximate composition of thigh meat. The total phenols content of thigh meat was in the range of 51.29 to 83.03 mg of *Gallic* acid equivalent/kg of meat at d 0 of storage.

The thigh from chickens fed the HEM supplement (particular 7.5 g/kg HEM diet) showed significantly greater total phenols content than the thigh of chickens fed the control diet at d 0, indicating that the antioxidative activity in the thigh meat of broiler chickens can be increased by dietary HEM, which is likely because of polyphenolic compounds. Previous study by Nuutila *et al*. (2002) showed that high amounts of polyphenols, flavonoids, and fiber in garlic decreased the muscle's fat oxidation.

The crude ash had linear decrease with the addition of HEM in diets. Furthermore, the crude fat of thigh was also decreased in supplemented group significantly.

Reported, the dietary administration of essential oil extract (such as rosemary) to broilers resulted in a decrease in the lipid and cholesterol oxidation of broiler meat during storage for 9 d and increased muscle's fat (Lopez-Bote *et al.*, 1998), But, the broilers given HEM in our study did not show any significant difference for moisture, and crude protein of thigh muscle.

In physicochemical evaluation, it was observed that pH and WHC decreased following the administration of HEM. Park and Yoo (1999) showed that the dietary Chinese medicine by-products at levels of 40 and 80 g/kg decreased the pH of thigh muscle in broiler chicks. In contrast with our results, a study by Sallam *et al*. (2004) found higher pH values in various types of garlic-treated chicken sausage compared with controls. Likewise, Simitzis *et al*. (2008) reported that the pH of female lamb meat increased after supplementation with dietary oregano essential oil.

One explanation was that the greater pH of meat might have reflected different glycogen reserves preslaughter (Simitzis *et al.*, 2008) because the nutritional treatment influenced muscle glycogen levels in cattle (Tudor *et al.*, 1996). Generally, it was observed that meat with high pH has a high WHC (Tudor *et al.*, 1996), although this was not proven to be so in this study. The WHC of thighs decreased linearly following the administration of HEM, which it is not a positive point in meat preservation.

Overall, there is limited information on the sensory quality of meat after dietary administration of medicinal herbs to animals. In our study, there were no differences in cooking loss percentage between broiler thigh meat from the HEM and control dietary treatments. Cooking loss percentages were less variable within each treatment and among treatments. Generally, the samples were very tender, indicating that they would be highly acceptable to consumers (Schilling *et al.*, 2003). Cooking loss of 216.9 to 236.5 g/kg for 7.5 g/kg HEM and control, respectively, was lower when compared with the 330 and 310 g/kg reported for organic and broiler chickens (Castellini *et al.*, 2002) and higher than 190 to 230 g/kg found for the Thai indigenous chicken (Wattanachant *et al.*, 2004).

Results from sensory evaluation revealed that within textural parameters such as hardness, flavor and overall acceptability of thigh meat improved by HEM.

On the other hand, juiciness (the amount of perceived juices in the meat during chewing), which is very important by consumers because it is one of the major meat characteristics that influence eating quality (Carr *et al.*, 1999), and it was higher in birds that achieved HEM, although it caused no significant difference and consequently these animals showed high fibrousness.

The WHC and shear force values can be used to determine if meat products vary in color, texture, and firmness or texture by measuring the variability in total cutting force (Kim *et al.*, 2009). Simitzis *et al*. (2008) reported that dietary oregano essential oil did not influence the shear force and sarcomere length in lamb meat. In different species, it has been established that the values of shear force increased with age due to an increase in the hardness of the connective tissue and also due to an increase in the collagen cross-linking (Fletcher, 2002).

Naveena and Mendiratta (2004) also asserted a decrease in shear force values with increasing amount of ginger extract in diet. Similarly, dietary medicinal herb increased the WHC of pork, but no difference was found in color, marbling, and firmness by a sensory test (Chen *et al.*, 2008). Because of that all samples from supplemented treatments in our study were more liked by consumers, they have given the highest acceptability score to supplemented groups. The highest score were given to 2.5 g/kg HEM by consumers and the lowest point was given to 0.0 g/kg additive. So these results indicated that feeding broilers with HEM at a concentration of 2.5 g/kg resulted in maximum effects on the sensory quality of chickens thigh.

Likewise, the TBARS values had also some differences on thigh meat that were stored for 0 and 1 d (2°C) and values than those that were reported by these researchers for samples that were stored for 5 to 10 d (Ang and Lyon, 1990). Results from our study were different from the study referenced above which is likely because the lipid percentage in the thighs was different in the referenced study. Some spices and herbs contain several compounds, mainly polypehnols (flavonoids, hydrolysable tannins, proanthocianidins, phenolic acids, phenolic terpenes) and some vitamins (E, C and A), which have antioxidant activities, so they can extend the shelf life and improve the quality of meat products (Liu *et al.*, 1992; Botsoglou *et al.*, 2002).

Phenolic compounds present in natural plant oils react with lipid and hydroxyl radicals and convert them into stable products (Yanishlieva-Maslarova, 2001). Simitzis *et al*. (2008) reported that phenolic compounds in oregano essential oil were absorbed and entered the systemic circulatory system after ingestion and had significant antioxidant activities in lamb meat. Overall, results from TBARS testing reveals that thigh meat from broilers that were fed 7.5 g/kg HEM may be less susceptible to oxidation than thigh meat from broilers fed with the control and under level diets. In our study, dietary HEM appeared to delay the lipid oxidation of broiler chicken meat, because the TBARS values of 2.5 to 7.5 g/kg thigh meat did not increase significantly during storage, whereas that of control thigh meat increased significantly.

It was shown that the dietary administration of rosemary and sage essential oil extract to broilers resulted in a decrease in the lipid and cholesterol oxidation of broiler meat during storage for 9 days (Lopez-Bote *et al.*, 1998). Likewise, supplementing turkeys with oregano extract increased the oxidative stability and retention of α-tocopherol in long-term frozen-stored turkey meat (Botsoglou *et al.*, 2003). However, it is still unclear whether the antioxidants consumed can be incorporated into fatty tissues in the same form as when the fat is stabilized in vitro (Vichi *et al.*, 2001). In present research the total phenols content of thigh meat from chickens fed HEM (2.5 to 7.5 g/kg diets) were significantly greater than that of chicken fed the control diet, indicating that phenolic compounds from HEM could prevent thigh meat from oxidizing. However, other compounds or mechanisms may be responsible for this antioxidant activity.

Also, further detailed studies are required to explain the possible mechanism of catalase activity with HEM supplementation. The overall antioxidant concentration in liver homogenates was higher in the group fed with HEM. In general, the antioxidant data suggest that HEM supplementation stimulated the antioxidant system in the liver for counteracting the oxidative damage caused by various agents. The low level of peroxides in the groups fed a combination of herbs probably indicated minimum cell damage in the liver because of adsorbed oxidation factors in the gut and tissue of birds in this age.

Moreover it was shown that many active components of herbs and spices can prevent lipid peroxidation through quenching free radicals or through activation of antioxidant enzymes like SOD, catalase, glutathione peroxidase and glutathione reductase (Botsoglou *et al.*, 2002, 2003).

Generally, the study asserts that the inclusion of HEM (7.5 g/kg) improved the overall antioxidant status and liver SOD activity and partially protected it against adverse effects of lipid peroxides, suggesting that higher levels of HEM may be required for maximum efficacy. Furthermore, in aspect of meat taste or hedonic, because of the HEM in level of 2.5 g/kg had the highest acceptability among panelists; we propose this protocol in necessary situations.
